# Effects of Patient Portal Message Framing on Treatment Preferences and Expectations for Degenerative Meniscus Tears: Randomized Exploratory Cross-Sectional Survey Study

**DOI:** 10.2196/92583

**Published:** 2026-05-12

**Authors:** Muzamil Ahmad, Annabella Jensen, Isabella Strickler, Ramon Arza, Matthew D Ramey, Conor Dolson, Jeremy Policht, Yzen Al-Marrawi, Leonardo Cavinatto

**Affiliations:** 1 Oakland University William Beaumont School of Medicine Rochester, MI United States; 2 Department of Orthopedic Surgery Corewell Health William Beaumont University Hospital Royal Oak, MI United States

**Keywords:** health literacy, decision-making, shared, communication, patient portals, arthroscopy, meniscus, knee injuries

## Abstract

**Background:**

Degenerative meniscus tears are common in middle-aged and older adults, and current guidelines favor nonoperative care. As patients increasingly turn to portal systems to view imaging results and communicate with their physicians, patient-facing wording may shape downstream treatment preferences and expectations.

**Objective:**

This study aimed to determine whether subtle differences in physician message framing about an identical degenerative meniscus tear influence preferred management, expectations for improvement with conservative therapy, and satisfaction when a physician recommends a different plan.

**Methods:**

A cross-sectional 37-question survey was developed de novo and distributed in January 2026 to lay adults in the United States (≥18 years) recruited via Amazon Mechanical Turk. Respondents were presented with a standardized vignette of an adult aged 60 years with knee pain due to a degenerative meniscus tear. Participants were randomized in a 1:1:1 fashion into 3 physician portal message framing groups: neutral, degenerative, and damage. Outcomes were the preferred next step in treatment, expected improvement with physical therapy, and retained satisfaction under physician-respondent disagreement. Chi-square and Fisher exact tests were used to compare categorical variables. Multivariable logistic regression analyses were used to assess associations between framing groups.

**Results:**

Of the 266 completed responses, 195 (73.3%) were included for analysis (neutral: n=67, 34.4%; degenerative: n=63, 32.3%; damage: n=65, 33.3%). Treatment preferences differed significantly across groups (*χ*^2^_2_=6.1; *P*=.047), and the damage group was significantly more likely to prefer aggressive interventions (odds ratio 2.43, 95% CI 1.17-5.06; *P*=.02). Expectations for physical therapy success differed significantly (*χ*^2^_4_=12.3; *P*=.02), with the damage group being most pessimistic about conservative care. Retained satisfaction under physician-respondent disagreement did not differ by framing group (*χ*^2^_6_=6.7; *P*=.35) but did differ significantly by initial treatment preference (*P*=.03) and was the lowest among respondents preferring steroid injection.

**Conclusions:**

In this exploratory investigation, subtle differences in physician portal message framing regarding a magnetic resonance imaging impression of a degenerative meniscus tear were associated with shifts in treatment preferences and confidence in conservative care. These findings suggest that brief physician portal communications may be associated with shifts in hypothetical patient expectations and treatment preferences before clinical counseling occurs.

## Introduction

Knee pain is a leading cause of disability in middle-aged and older adults [1]. During clinical evaluation of the knee, magnetic resonance imaging (MRI) is often used to investigate suspected soft tissue pathology. As MRI availability and application continues to expand, clinicians may inevitably encounter meniscus tears, whether they be multifactorial or degenerative on presentation. Previous literature has demonstrated that the prevalence of degenerative meniscal damage rises substantially with age and may present without any knee pain [2]. Despite this high prevalence of degenerative meniscal findings among older patients, numerous trials have demonstrated little to no benefit with operative management over nonoperative care [3,4]. As a result, current guidelines regarding management of meniscus tears in older patients are primarily nonoperative [5]. However, patient beliefs about what a “tear” means could potentially alter treatment preferences and satisfaction with conservative care. Previous literature reports that patients cite MRI results as the key driver in the decision-making process for the management of degenerative meniscus tears and that surgery is the definitive and quicker approach compared to physical therapy (PT) [6].

At the same time, the growth of the electronic medical record has transformed physician-patient communication, and patients often have access to their imaging results through patient portals before any interaction with their physicians [7]. As such, radiology reports, which are written for medical audiences, may contain terminology that could be confusing or anxiety provoking to a patient, altering their perceived level of severity and urgency [8]. Specifically in musculoskeletal imaging, pathological terminology can carry strong implied meaning to a lay audience (ie, “tear,” “degeneration,” “damage,” and “complex”), potentially being interpreted as more severe with a higher need for operative repair even when nonoperative management is the established norm.

Existing literature across various musculoskeletal pathologies has shown that diagnostic labels on imaging results can influence patient beliefs and perceived severity. In prior work, patients interpreted “wear and tear” labels as evidence of mechanical degradation and indicated concerns that continued activity would worsen joint “damage” [9,10]. Other studies have shown that pathologizing diagnostic labels shift perceived needs toward aggressive intervention, increase negative expectations, and reduce engagement in beneficial conservative therapy [11,12]. Although the effects of imaging impression labels are increasingly recognized, little is understood about the effect of physician-patient communication regarding meniscal tears in shaping downstream treatment preferences, expectations, and satisfaction.

Therefore, we conducted a randomized cross-sectional study to investigate whether subtle differences in physician messaging regarding a degenerative meniscus tear affect preferred next steps in management, expectations regarding improvement with conservative therapy, and overall satisfaction under patient-physician disagreement. We hypothesized that more pathologizing language (ie, “damage”) would increase preferences for aggressive interventions and lower confidence in conservative management, with higher rates of dissatisfaction under patient-physician disagreement compared to neutral or degenerative framing.

## Methods

A cross-sectional, 37-question survey was developed de novo to evaluate whether patient-facing physician messaging influences treatment preferences, expectations, and satisfaction.

### Survey Content

A 37-question instrument was developed de novo combining multiple-choice questions and numeric free-text responses to capture the study objectives (see Multimedia Appendix 1 for the survey instrument). The instrument was revised and pilot-tested among the study team for functionality prior to distribution. Initial questions screened for respondents who had any active or prior health care–related work experience. Further questions collected baseline demographics. Demographic information included age, sex, race, insurance status, and educational level. Health literacy was measured by polling the frequency of needing assistance in understanding health material. Prior knee-related history (history of knee pain, knee MRI, knee PT, or knee surgery) and general risk tolerance were also collected.

Subsequent sections presented randomized scenarios of a fixed knee injury and brief communications with a physician and then collected respondent preferences, expectations, and satisfaction regarding the management of this knee injury. Education regarding the meniscus and MRI function was provided to participants alongside the standardized vignette before formal questioning began.

### Study Population and Recruitment

A nonclinical sample was selected to isolate how patient-facing portal wording shapes initial preferences before specialist counseling, reducing confounding from an orthopedic clinical sample that may already be exposed to specialist counseling and, thereby, may influence responses.

Participants were recruited through Amazon Mechanical Turk (MTurk), a clinically validated online crowdsourcing marketplace tool in which “requestors” financially compensate “workers” for completing human intelligence tasks (HITs), which commonly include health-related survey-based studies [13]. To enhance the quality of respondents, participation was restricted to workers with a prespecified HIT rating demonstrating a history of high-quality responses (≥95%). Inclusion criteria were age of 18 years or above, residence in the United States, and an MTurk HIT rating of 95% or higher. These criteria were enforced using MTurk worker qualifications. Exclusion criteria were respondents indicating any current or prior health care–related work experience and failure to pass embedded attention checks throughout the survey. Health care work experience was excluded to reduce bias from existing medical knowledge that could alter the reported outcomes. Respondents who failed any attention check were excluded to improve data quality. To avoid missing data, all survey questions required a response to advance and submit.

All survey questions were administered using an online survey platform (Qualtrics XM; Qualtrics International Inc) with embedded security checks enabled. The survey required approximately 3 to 10 minutes to complete. At the end of the Qualtrics survey, each participant was presented with a completion code, which they were required to enter into the MTurk interface to receive compensation. Completion codes were matched between Qualtrics and MTurk to ensure that each response corresponded to a unique, valid respondent. Incomplete survey responses or entries with invalid codes were rejected and did not receive compensation.

### Survey Instrument and Vignette

All participants were presented with the same standardized clinical vignette:

In this scenario, imagine you are a 60-year-old adult who has had knee pain and swelling for nearly 6 weeks. The symptoms started after a twisting movement on that leg and are felt most on the inner side of the knee. The pain is worse when bending the knee, going up or down stairs, and when squatting. You can still walk, but you have had to limit the amount of exercise you do because of the pain and swelling. The knee has not locked in place (never gets stuck when trying to fully straighten the leg), but it sometimes clicks. You were referred to a joint specialist doctor who ordered an MRI of your knee. The MRI report comes back and reads:

MRI Impression: “Horizontal tear of the posterior horn of the medial meniscus.”

**Figure 1:**
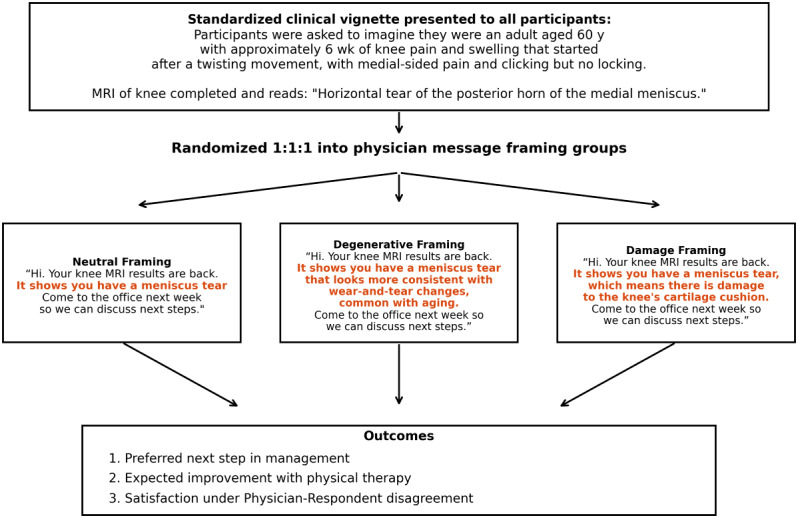
Study design and physician message framing interventions highlighted in bold red text. MRI: magnetic resonance imaging.

MRI impressions were identical for all participants to hold pathology constant and simulate real-world patient access to MRI results in patient portal systems. Following this, participants were randomized in a 1:1:1 ratio into 3 separate framing groups: neutral, degenerative, and damage. After group randomization, participants were presented with a brief simulated portal message from the joint specialist using language specific to the assigned framing groups (Figure 1). Following this, participants’ preferences, expectations, and satisfaction were assessed.

### Primary and Secondary Outcomes

The primary outcomes were preferred next steps in management and perceived level of confidence in conservative therapy success. This was assessed by presenting a multiple-choice question of conservative (nonoperative or second opinion) and invasive (procedural) options: (1) start PT, (2) steroid injection, (3) rest and reassess, (4) surgery, and (5) seek a second opinion. Invasive options were surgery and steroid injection. Conservative options were PT, rest and reassess, and seeking a second opinion.

The secondary outcome was retained satisfaction in a follow-up scenario in which the joint specialist disagreed with the respondents’ preferred next step in management and whether satisfaction had any association with a respondent’s initial preferences. Satisfaction was measured on an ordinal response scale (“very dissatisfied,” “dissatisfied,” “neutral,” “satisfied,” and “very satisfied”).

### Statistical Analysis

Descriptive statistics were presented as means and SDs. Categorical variables, including Likert distributions, were compared using chi-square and Fisher exact tests. Pairwise comparisons were reported using odds ratios (ORs) with 95% CIs. Multivariable logistic regression analysis was performed to assess whether associations between framing groups persisted after adjusting for baseline characteristics. A *P* value of .05 or less was considered statistically significant. Given the exploratory nature of this study and the limited sample size, formal correction for multiple comparisons was not performed. Secondary pairwise comparisons and post hoc stratified analyses were considered hypothesis generating and should be interpreted cautiously.

Exploratory post hoc sensitivity analyses were performed stratifying by prior knee MRI status. In each stratum, multivariable logistic regression models were used for preference for invasive management and confidence in PT. Formal interaction testing was used to assess whether prior knee MRI status carried associations between framing groups and outcomes.

### Ethical Considerations

Per institutional policy, this study was determined not to constitute human subjects research and therefore did not require formal institutional review board review (Multimedia Appendix 2). All survey responses were anonymous and contained no identifying information. Participants received US $0.50 in compensation upon completion.

## Results

### Overview

In total, 266 American adults completed the survey. Of these 266 participants, 67 (25.2%) were excluded due to indicating prior or current health care–related work experience, and 4 (1.5%) were excluded due to failing the embedded attention checks. No data were missing as all survey questions required a response. A total of 195 respondents were included for analysis: the neutral framing group included 67 (34.4%) respondents, the degenerative framing group included 63 (32.3%) respondents, and the damage framing group included 65 (33.3%) respondents.

Participant characteristics were highly similar across groups (Table 1). Mean respondent age was approximately the same across all 3 groups (neutral: 33.3, SD 4.4 years; degenerative: 35.0, SD 6.3 years; damage: 33.5, SD 3.8 years). All 3 groups were predominantly male (155/195, 79.5%) and White (187/195, 95.9%), with the vast majority holding a bachelor’s degree or higher (187/195, 95.9%). Prior knee-related experiences (prior knee pain of >4 weeks, prior knee MRI, prior knee PT, or prior knee surgery) were comparable across groups, and each group scored relatively similarly with regard to self-reported risk tolerance (*P*=.13; Table 1).

**Table 1:** Baseline respondent characteristics overall and by framing group (N=195)^a^.

Characteristics	Overall	Neutral (n=67)	Degenerative (n=63)	Damage (n=65)	*P* value
Age (y), mean (SD)	33.9 (5.0)	33.3 (4.4)	35.0 (6.3)	33.5 (3.8)	.11
Male sex, n (%)	155 (79.5)	52 (77.6)	52 (82.5)	51 (78.5)	.76
White race, n (%)	187 (95.9)	62 (92.5)	62 (98.4)	63 (96.9)	.21
Bachelor’s degree or higher, n (%)	187 (95.9)	65 (97.0)	60 (95.2)	62 (95.4)	.85
Insured, n (%)	191 (97.9)	65 (97.0)	62 (98.4)	64 (98.5)	.80
Sometimes, often, or always needed help reading medical materials, n (%)	182 (93.3)	63 (94.0)	57 (90.5)	62 (95.4)	.52
Prior knee pain for >4 wk, n (%)	86 (44.1)	31 (46.3)	28 (44.4)	27 (41.5)	.86
Prior knee MRI^b^, n (%)	92 (47.2)	30 (44.8)	31 (49.2)	31 (47.7)	.88
Prior knee physical therapy, n (%)	113 (57.9)	42 (62.7)	36 (57.1)	35 (53.8)	.58
Prior knee surgery, n (%)	52 (26.7)	20 (29.9)	15 (23.8)	17 (26.2)	.73
Risk tolerance (1-10), mean (SD)	5.61 (3.40)	6.16 (3.21)	5.67 (3.41)	4.97 (3.54)	.13

^a^Participants were adults in the United States recruited via Amazon Mechanical Turk in January 2026. Categorical variables are presented as frequencies and percentages and were compared using chi-square and Fisher exact tests where appropriate. Continuous variables are presented as means and SDs and were compared using 1-way ANOVA.

^b^MRI: magnetic resonance imaging.

### Management Preferences and Expectations

Following the presentation of the physician portal message, respondents were polled on their preferred next steps in management. The groups varied significantly in the overall distribution of their treatment preferences (*P*=.047). The damage group had the highest rate of preference for invasive management (ie, surgery or intra-articular injection; 48/65, 73.8%) compared to the neutral (36/67, 53.7%) and degenerative (37/63, 58.7%) groups and was significantly more likely to prefer invasive management compared to the neutral group (OR 2.43, 95% CI 1.17-5.06; *P*=.02). The neutral group was most likely to seek conservative management (ie, rest and reassess, PT, or seeking a second opinion; 31/67, 46.3%), followed by the degenerative group (26/63, 41.3%) and damage group (17/65, 26.2%; Table 2).

**Table 2:** Preferred management by framing group^a^.

Framing	Conservative management, n (%)	Invasive management, n (%)
Neutral (n=67)	31 (46.3)	36 (53.7)
Degenerative (n=63)	26 (41.3)	37 (58.7)
Damage (n=65)	17 (26.2)	48 (73.8)

^a^Conservative options included physical therapy, rest and reassess, and second opinion. Invasive options included steroid injection and surgery. Between-group distributions were compared via chi-square test (*χ*^2^_2_=6.1; *P*=.047). Pairwise comparisons were assessed via Fisher exact tests (damage vs neutral: odds ratio [OR] 2.43, 95% CI 1.17-5.06, and *P*=.02; degenerative vs neutral: OR 1.23, 95% CI 0.61-2.45, and *P*=.60).

**Figure 2:**
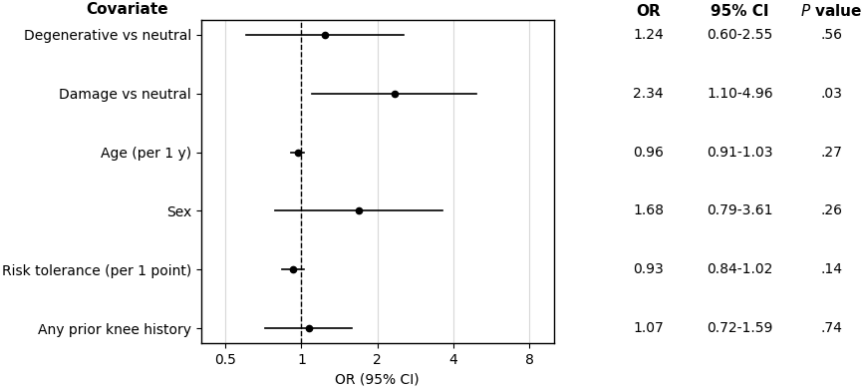
Multivariable logistic regression of invasive management preference. Odds ratios (ORs) and 95% CIs from a multivariable logistic regression model evaluating factors associated with preference for invasive management (steroid injection or surgery) vs conservative management (physical therapy, rest and reassess, or second opinion), with neutral framing held as the reference. Covariates included age, sex, risk tolerance, and prior knee history. Any prior knee history included knee pain of more than 4 weeks, knee magnetic resonance imaging, knee physical therapy, and knee surgery.

In multivariable logistic regression analysis, the damage group remained independently associated with greater odds of preferring invasive management compared with the neutral group (OR 2.34, 95% CI 1.10-4.96; *P*=.03), whereas the degenerative group was not significantly associated with invasive preference (OR 1.24, 95% CI 0.60-2.55; *P*=.56; Figure 2).

Between-group expectations regarding confidence in conservative therapy success varied significantly (*χ*^2^_4_=12.3; *P*=.02). In the damage group, respondents were most pessimistic about conservative therapy and more frequently indicated “little improvement” expected from PT (16/65, 24.6%) compared to the neutral (7/67, 10.4%) and degenerative (9/63, 14.3%) groups, whereas the neutral and degenerative groups more frequently indicated “moderate” and “great” improvement with PT over the damage group (neutral: 60/67, 89.6%; degenerative: 54/63, 85.7%; damage: 49/65, 75.4%; Table 3).

**Table 3:** Expected improvement with physical therapy by framing group^a^.

Expectation	Neutral (n=67), n (%)	Degenerative (n=63), n (%)	Damage (n=65), n (%)
No improvement	0 (0)	0 (0)	0 (0)
Little improvement	7 (10.4)	9 (14.3)	16 (24.6)
Moderate improvement	34 (50.7)	43 (68.3)	32 (49.2)
Great improvement	26 (38.8)	11 (17.5)	17 (26.2)

^a^Between-group distributions were assessed via chi-square test (*χ*^2^_4_=12.3; *P*=.02).

**Figure 3:**
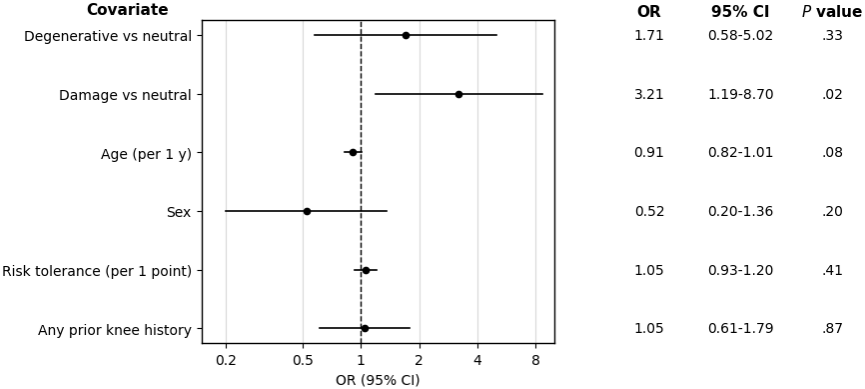
Multivariable logistic regression analysis of pessimism toward physical therapy. Odds ratios (ORs) and 95% CIs from a multivariable logistic regression model evaluating factors associated with pessimism regarding improvement with physical therapy. Neutral framing is held as the reference. Covariates included age, sex, risk tolerance, and prior knee history. Any prior knee history included knee pain of more than 4 weeks, knee magnetic resonance imaging, knee physical therapy, and knee surgery.

In multivariable logistic regression analysis for expectations regarding improvement with PT, respondents in the damage group had greater odds of expecting little improvement with PT compared with the neutral group (OR 3.21, 95% CI 1.19-8.70; *P*=.02), whereas the degenerative group did not differ significantly from the neutral group (OR 1.71, 95% CI 0.58-5.02; *P*=.33; Figure 3).

### Post Hoc Sensitivity Analysis by Prior Knee MRI

In exploratory post hoc analyses stratifying by prior knee MRI status, the observed direction of the framing effect on the primary outcomes remained similar across strata. Among respondents with prior knee MRI (92/195, 47.2%), damage framing was significantly associated with greater odds of preferring invasive management (OR 3.17, 95% CI 1.04-9.67; *P*=.04), whereas degenerative framing was not (OR 1.41, 95% CI 0.49-4.09; *P*=.52). Damage framing was also significantly associated with greater odds of expecting little improvement with PT (OR 5.14, 95% CI 1.16-22.80; *P*=.03), whereas degenerative framing was not (OR 2.30, 95% CI 0.46-11.55; *P*=.31).

Among respondents without prior knee MRI (103/195, 52.8%), primary outcome associations by framing group were directionally similar, but damage framing did not reach statistical significance for invasive management preference (OR 1.84, 95% CI 0.61-5.56; *P*=.28) or expectations regarding PT (OR 1.91, 95% CI 0.46-7.95; *P*=.37). On formal interaction testing, no significant effect modification by prior knee MRI status was present for invasive management preference (*P*=.76) or expectations regarding improvement with PT (*P*=.72).

### Physician-Patient Disagreement and Rated Satisfaction

When presented with the scenario in which the treating physician disagreed with the respondent’s preferred management, overall physician satisfaction ratings did not differ significantly between groups (*χ*^2^_6_=6.7; *P*=.35).

While satisfaction under treatment discordance did not differ across groups, it did vary significantly by respondents’ preferred next step (*χ*^2^_12_=23.0; *P*=.03). This finding was also consistent when satisfaction was dichotomized as “satisfied” or “very satisfied” vs not satisfied (*χ*^2^_4_=11.7; *P*=.02). Of all respondents, only 40% (26/65) of those who preferred steroid injection indicated that they would retain any form of satisfaction with their joint specialist under disagreement. This was contrary to the retained satisfaction rates of those who preferred surgery (38/56, 67.9%), PT (26/40, 65%), second opinion (10/16, 62.5%), and rest and reassess (11/18, 61.1%; Table 4).

**Table 4:** Retained satisfaction under physician disagreement by preferred treatment^a^.

Preferred management next step	Satisfied, n/N (%)
Meet with specialist to ask about surgery	38/56 (67.9)
Start physical therapy first	26/40 (65)
Seek a second opinion immediately	10/16 (62.5)
Rest and reassess later	11/18 (61.1)
Ask about steroid injection	26/65 (40)

^a^Between-group distributions were assessed via chi-square test (*χ*^2^_12_=23.0; *P*=.03). Satisfaction was dichotomized as “satisfied” or “very satisfied” vs not satisfied (*χ*^2^_4_=11.7; *P*=.02). For interpretability, “satisfied” was dichotomized as “satisfied” and “very satisfied.”

In understanding the discrepancy in retained satisfaction rates between those who preferred steroid injection and those who preferred surgery, 2 invasive options, between-group analysis of primary drivers of treatment selection showed that those who indicated a preference for steroid injection significantly weighed the speed of pain relief and return to normalcy as the most important drivers of their treatment selection compared to those who preferred surgery (steroid injection: 45/65, 69.2%; surgery: 28/56, 50%; *P*=.04; OR 2.25, 95% CI 1.07-4.73; Table 5). No other significant associations between drivers and treatment preferences were observed.

**Table 5:** Primary motivators of treatment selection among respondents preferring steroid injection vs surgery^a^.

Motivator	Steroid injection (n=65), n (%)	Surgery (n=56), n (%)	*P* value	OR^b^ (95% CI)
I want the fastest pain relief and return to normalcy.	45 (69.2)	28 (50)	*.04* ^c^	*2.25 (1.07-4.73)*
The word “tear” sounded serious.	15 (23.1)	11 (19.6)	.67	1.23 (0.51-2.95)
Specialist made it seem urgent.	20 (30.8)	16 (28.6)	.84	1.11 (0.51-2.43)
Specialist made it seem less serious/common/age-related.	30 (46.2)	27 (48.2)	.86	0.92 (0.45-1.88)
Felt specialist implied surgery recommended next step.	27 (41.5)	23 (41.1)	>.99	1.02 (0.49-2.11)
I prefer to try the least invasive option first.	9 (13.8)	6 (10.7)	.78	1.34 (0.45-4.03)
I worry PT won’t work/will take too long.	2 (3.1)	7 (12.5)	.08	0.22 (0.04-1.12)
I worry knee could get worse if I delay definitive surgery.	13 (20)	17 (30.4)	.21	0.57 (0.25-1.32)
I worry about surgical complications.	5 (7.7)	6 (10.7)	.75	0.69 (0.20-2.41)
Cost/insurance concerns influenced my choice.	3 (4.6)	5 (8.9)	.47	0.49 (0.11-2.17)

^a^Pairwise comparisons were assessed via Fisher exact tests.

^b^OR: odds ratio.

^c^Reached statistical significance (*P*<.05).

## Discussion

### Principal Findings

#### Overview

In this randomized cross-sectional study, lay adults in the United States were polled to investigate whether patient-facing portal message framing about an identical MRI report of a horizontal medial meniscus tear influences preferred management, confidence in conservative therapy, and satisfaction when the physician disagrees with that preference. Overall treatment preferences differed significantly across the neutral, degenerative, and damage framing arms. Participants exposed to damage framing were significantly more likely to prefer invasive management compared with the other groups, supporting our hypothesis that more pathologizing language may have the potential to shift patient preferences toward more aggressive interventions. Regression analyses supported the primary findings, suggesting that the association among damage framing, invasive treatment preference, and pessimism toward conservative therapy in this sample is reproducible even when adjusting for baseline characteristics. In exploratory post hoc analyses for prior knee MRI status, these associations similarly remained directionally consistent. These findings show that pathologizing language (damage) for degenerative meniscus tears may be associated with an inclination toward invasive interventions and lower expectations for conservative management. Additionally, the observations align with existing literature demonstrating that the presentation method of health information can significantly influence patient decision-making and risk perception in medical contexts [7-12]. The framing effect, a robust cognitive bias in decision science, has been shown to alter choices based on semantic description of equivalent information and is relevant in medical decisions where language can shape preferences and perceived risks or benefits [14]. Although some outcomes did not reach statistical significance in this sample, the consistency of the directional trends suggests that these findings warrant further investigation in real-world clinical populations.

#### Clinical Framing and Patient Expectations

The distinct directional trends observed, with higher preferences for surgery or steroid injection in the damage arm and greater expectations for conservative therapy in the neutral and degenerative arms, may be explained through well-described framing and attribute effects. The classic literature on frame-dependent decision-making finds that individuals respond differently to equivalent health information framed in terms of losses vs gains or severity vs normalcy, which in turn influences risk tolerance and choice behavior [15]. In our study, describing a meniscal tear as “damage” (implying cartilage injury) could have served as a negative frame that increased perceived severity and encouraged preferences for more aggressive interventions.

This phenomenon is not limited to cognitive exercises; clinicians’ wording during clinical encounters can shape patient expectations, emotional responses, and subsequent preferences for care. Prior qualitative research on meniscal pathology highlights that patients’ perceptions of MRI findings strongly influence their treatment decisions, with imaging often serving as a key driver in decision-making and greater worry about structural “damage” leading to preferences for definitive interventions [6]. Our findings extend this literature by demonstrating that such influence can occur downstream of MRI findings at the level of a brief portal-style message before any face-to-face clinical discussion occurs.

Notably, expectations regarding PT effectiveness differed significantly between groups, further supporting the idea that language framing influences not only treatment preferences but also beliefs about treatment efficacy. Participants in the damage group were significantly more pessimistic about the likelihood of improvement with PT, whereas respondents in the neutral and degenerative groups were more likely to expect moderate to great improvement with PT. This finding may be clinically relevant because patient expectations can influence perceptions of treatment effectiveness and willingness to pursue conservative care. Prior literature has demonstrated associations between treatment expectations and health behaviors across musculoskeletal conditions [16]. However, this study did not evaluate real-world treatment adherence, PT participation, or clinical outcomes, and any downstream behavioral implications remain speculative. Given the hypothetical nature of this study design, further research in real-world orthopedic populations is needed to determine whether these expectation differences translate into actual treatment decisions or rehabilitation behaviors.

#### Physician Communication and Satisfaction

Although respondent satisfaction with the treating physician under hypothetical discordance did not significantly differ by framing group, it did differ significantly by initial treatment preference.

High patient satisfaction ratings underscore the importance of clinician communication quality, resonating with extensive evidence that patient-centered communication, where demonstrating empathy, respect, and shared decision-making, is associated with higher satisfaction and better adherence to treatment plans [17]. Research in a wide range of clinical contexts demonstrates that communication behaviors, including clarity of information, inclusive language, and perceived responsiveness, strongly influence patients’ perceptions and evaluation of care [18].

Furthermore, the literature suggests that adequate physician-to-patient information delivery enhances patient satisfaction with treatment decisions and trust in clinicians. In chronic conditions such as ulcerative colitis, greater exchange of disease and treatment information correlates with higher satisfaction and trust, highlighting the broader importance of communication content and style in satisfaction beyond specific framing effects [19]. Our finding that satisfaction varied more strongly by a respondent’s initial treatment preference than by framing group further highlights the importance of understanding how early expectations are formed. In particular, respondents who preferred steroid injection demonstrated significantly lower retained satisfaction under disagreement with the treating physician compared with those preferring surgery or conservative management, and this subgroup more strongly prioritized rapid symptom relief and return to function as the main drivers of their treatment choice. While, in this sample, the framing group did not significantly influence satisfaction with the treating physician under disagreement, specific interventions did. Therefore, if initial message framing primes patient inclinations toward specific interventions, it may indirectly influence downstream satisfaction even when clinical recommendations remain unchanged, although this is a hypothesis-generating inference.

### Implications for Shared Decision-Making

Our findings also complement broader research emphasizing the role of communication in shared decision-making. Decision aids and structured communication strategies have been shown to support patient engagement by presenting options in a balanced way and facilitating deliberation. Such tools aim to reduce unintended framing bias and help patients understand the implications of potential outcomes [20]. This aligns with the principle that framing should be used carefully to support, not skew, patient decisions.

These implications are particularly relevant in modern orthopedic practice, where patients increasingly access imaging reports and brief physician messages through electronic health portals before in-person discussion. Early exposure to framed language may shape expectations before shared decision-making formally begins, potentially influencing how patients interpret subsequent recommendations. Our findings suggest that early exposure to subtly pathologizing language may prime patients to view their condition as more severe, develop lower confidence in conservative care, and form preferences for invasive intervention before shared decision-making formally begins. Additionally, given ongoing concerns regarding overuse of invasive procedures for degenerative meniscal pathology, communication framing may represent an underrecognized contributor to patient demand for low-value interventions. Even modest shifts in patient expectations driven by language could have meaningful downstream effects on use at scale.

### Limitations and Future Directions

This study has several limitations that should be considered. First, the sample was limited to lay adults in the United States and may not represent the behaviors of orthopedic clinic populations in other settings; respondents were located in the United States and predominantly male, White individuals, and highly educated. As a result, the observed findings may not reflect real-world clinical populations both in the United States and elsewhere, limiting the generalizability of these findings. Second, management preferences were assessed using a hypothetical scenario rather than lived clinical experiences, and decision-making may differ when individuals are personally affected by a condition. Third, the magnitude of influence on patient behaviors that portal-style messaging possesses may vary depending on how a specific message is framed and could have many different impacts on attitudes and behaviors that fall outside the scope of this study. Fourth, while the exclusion of respondents who indicated any form of health care experience was done to increase internal validity, it may have also reduced generalizability by producing a more curated lay sample than would be expected in real-world clinical practice. Fifth, nearly half (92/195, 47.2%) of the respondents reported a prior knee MRI despite the relatively younger age of the study population. The plausible explanations for this are hypothesis generating in nature and could reflect selection bias in which participants with prior musculoskeletal history were drawn to the title and description of the survey on the MTurk web interface, limiting generalizability and necessitating a post hoc sensitivity analysis stratified by prior knee MRI status. Sixth, this study was designed around a hypothetical and simulated clinical vignette of a patient aged 60 years through which the study intervention was presented to participants who were relatively young. Therefore, the observed findings may not be reflective of the real-world decision-making of actual middle-aged and older patients, who would be most likely to experience this pathology, and may not capture the views of older orthopedic patients, who may possibly respond differently to the framing effects and treatment preferences. Seventh, while the primary aim of this study was to assess framing effect on invasive vs conservative treatment preferences, collapsing steroid injection and surgery into 1 category could have obscured additional behavioral and decision-making distinctions. Eighth, as exclusions were made after recruitment through health care experience screening and failed attention checks, subgroup sizes for each framing type, while similar in size, were not exactly even, and this could have influenced overall balance. Ninth, the language used in the damage framing group could reasonably have influenced the observed findings as describing “damage to the knee’s cartilage cushion” may have introduced both a pathologizing word (“damage”) and a structural association, possibly influencing perceived severity. Finally, although the study was randomized, the individual group sizes could have limited statistical power to detect additional smaller effects.

Accordingly, future research is needed to validate these findings, which are exploratory and hypothetical in nature, ideally replicating this study using a real-world clinical patient population. Further work should also seek to qualitatively characterize how patients interpret commonly used orthopedic terms such as “tear,” “degeneration,” and “damage,” which may inform the development of clearer, more patient-centered communication strategies.

### Conclusions

In this exploratory cross-sectional study, subtle differences in physician portal message framing regarding an MRI impression of a degenerative meniscus tear were associated with shifts in treatment expectations and, in some cases, increased preferences for more aggressive management and decreased confidence in guideline-concordant conservative therapy. These findings suggest that physician portal language may influence hypothetical patient expectations and treatment preferences prior to clinical counseling. Future studies in real-world orthopedic populations are needed to determine whether these observed framing effects translate to actual treatment decisions.

## Data Availability

Data are available upon request to the corresponding author.

## Appendix

Multimedia Appendix 1 Survey instrument.

Multimedia Appendix 2 Institutional review board review exemption policies from Oakland University William Beaumont School of Medicine.

## Abbreviations

HIT: human intelligence task
MRI: magnetic resonance imaging
MTurk: Amazon Mechanical Turk
OR: odds ratio
PT: physical therapy
