# Supplementary material for: Effects of Patient Portal Message Framing on Treatment Preferences and Expectations for Degenerative Meniscus Tears: Randomized Exploratory Cross-Sectional Survey Study
Source: JMIR Form Res. 2026 May 12;10:e92583. doi: 10.2196/92583 (PMC13213325; doi:10.2196/92583)
Supplement: Multimedia Appendix 2 [file formative_v10i1e92583_app2.docx]

**OAKLAND UNIVERSITY**

**INSTITUTIONAL REVIEW BOARD**

**NOT HUMAN SUBJECTS RESEARCH DETERMINATION GUIDANCE**

**RESEARCHER’S WORKSHEET ONLY**

**(DO NOT SUBMIT THIS FORM TO THE IRB)**

**IMPORTANT NOTES:**

- If researchers require a **“Not Research” or** “**Not Human Subject Research” determination letter** from the IRB for publication or presentation purposes, they must submit an “**Initial**” submission in the online system, Cayuse, and select the “**Not Research or Not Human Subject Research**” option in **Section 2- Type of Application**. The application will be reviewed administratively by the IRB Staff.
- The purpose of this document is to serve as a worksheet to assist researchers in determining if their proposed activity or project requires IRB review.
- If the project does not constitute “**Research**” or “**Human Subjects Research,**” it is not under the purview of the IRB. However, researchers are highly encouraged to consult with the IRB staff to confirm a not human subject research determination so that they do not end up conducting human subjects research without proper IRB approval. Conducting human subjects research without IRB approval or exemption constitutes a case of non-compliance. Data collected without IRB approval may not be used for publication or presentation.
- For questions, please contact Judette Haddad, PhD, IRB Manager at [haddad@oakland.edu](mailto:haddad@oakland.edu) or Kate Wydeven, IRB Specialist at [kwydeven@oakland.edu](mailto:kwydeven@oakland.edu)

**SECTION 1. ACTIVITIES THAT DO NOT CONSTITUTE HUMAN SUBJECTS RESEARCH or NOT RESEARCH**

A. ☐ **Course-related activities**: The project involves activities designed specifically for educational or teaching purposes where data are collected as part of the class exercise or course requirement but are not intended for use outside the classroom.

B. ☐ **Case Report**: The project is limited to a report about the experiences or observations of **three or less** individuals in which there was no research intervention and no intent to contribute to generalizable knowledge. **Note**: If the case report is a clinical report that involves the disclosure of Protected Health Information (PHI) as defined by the HIPAA Privacy Rule, HIPAA authorization/waiver must be first obtained. Contact the IRB staff for assistance.

C. ☐ **Program Evaluation/Quality Assurance/Quality Improvement Projects**: The project involves activities designed specifically to assess or improve performance within a department, university or classroom setting, or quality of care or efficiency of an institutional practice. The intention of the project is not to generate conclusions that can be applied universally, or outside the immediate environment where the project occurred.

D. ☐ **Oral History**: The project is limited to oral history activities, such as open ended interviews, that only document a specific historical event or the experiences of individuals without the intent to draw conclusions or generalize findings. **Note**: IRB review and approval is required when the oral history activities are intended to produce generalizable conclusions (e.g., activities that serve as data collection intended to test economic, sociological, or anthropological models/theories).

E. ☐ **Journalism/Documentary Activities**: The project’s activities are limited to investigations and interviews that focus on specific events, views, etc., and that lead to publication in any medium (including electronic) or documentary production, or are part of training that is explicitly linked to journalism. There is no intent to test a hypothesis. **Note**: IRB review and approval may be required when journalists conduct activities normally considered scientific research intended to produce generalizable knowledge (e.g., systematic research, surveys, and/or interviews that are intended to test theories or develop models).

F. ☐ **Research Using Publically Available Data**: The project is limited to analyzing publically available data. Publically available means that the information is shared without conditions on use. Data sets that require a fee to gain access to the data are not publically available. **Note**: IRB review and approval is required if the publically available data set includes identifiers or subjects can be uniquely identified through a combination of indirect identifiers.

G. ☐ **Research on Organizations**: Information gathering about organizations, including information about operations, budgets, etc. from organizational spokespersons or data sources. The project may not include identifiable private information about individual members, employees, or staff of the organization.

H. ☐ **Research on Decedents**: The projects involves data or biospecimens from deceased individuals and the data collected will not affect secondary individuals such as family members (e.g., genetic studies).

Note: If the project involves the use and/or collection of Protected Health Information (PHI), researchers must confirm certain criteria are met in accordance with the HIPAA Privacy Rule. If PHI will be used in the proposed project or activity involving decedents, confirm ALL the following criteria will be met:

1. ☐ the use or disclosure will be solely for research on the PHI of a decedents; **and**
2. ☐ the Principal Investigator has documentation of the death of the individual about whom the PHI is being sought; **and**
3. ☐ the PHI being sought is necessary for the research.

I. ☐ **De-identified Private Information**: The project is limited to the use of existing and/or prospectively collected de-identified private information provided ALL of the following criteria are met when applicable:

1. ☐ The private information was/is not collected specifically for the currently proposed research through an intervention or interaction with living individuals; **and**
2. ☐ The researcher can confirm that the use of private information is not in violation of the terms under which the information was originally collected; **and**
3. ☐ The researcher will only receive information that is fully de-identified. De-identified means that the identity of the individual cannot be readily ascertained or does not include any of the 18 identifiers defined in the HIPAA Privacy Rule. De-identified data cannot be linked back to individuals directly or through coding systems; **and**
4. ☐ The records/images/charts that are being collected for this project are NOT from individuals who are or will become recipients of an FDA regulated product or act as an control as directed by a research protocol and not by medical practice, and the results are to be submitted to FDA or held for inspection by the FDA.

J. ☐ **De-identified Human Biospecimens**: The project is limited to the use of existing and/or prospectively collected human biospecimens provided ALL of the following criteria are met:

1. ☐ The biospecimens were/are not collected specifically for the currently proposed research through an intervention or interaction with living individuals; **and**
2. ☐ The researcher can confirm that the use of the biospecimens is not in violation of the terms under which the biospecimens were originally collected; **and**
3. ☐ The researcher will only receive biospecimens that are fully de-identified. De-identified means that the identity of the individual cannot be readily ascertained or does not include any of the 18 identifiers defined in the HIPAA Privacy Rule. De-identified biospecimens cannot be linked back to individuals directly or through coding systems; **and**
4. ☐ Biospecimens are NOT being used to test the effectiveness of a medical device or as a control in an investigation of an investigational device and the results of the activity are to be submitted to the FDA or held for inspection by the FDA.

K. ☐ **Coded* Private Information and/or Human Biospecimens:** The project is limited to the use of existing and/or prospectively collected coded private information and/or human biospecimens provided ALL of the following criteria are met when applicable:

1. ☐ The private information or biospecimens were/are not collected specifically for the currently proposed research through an intervention or interaction with living individuals; **and**
2. ☐ The researchers (including anyone involved in conducting the research) cannot readily ascertain the identity of the individual(s) to whom the coded private information or biospecimens pertain because, for example:

(a) ☐ The researchers and the holder of the key enter into an agreement prohibiting the release of the key to the researchers under any circumstances, until the individuals are deceased;

(b) ☐ There are IRB-approved written policies and operating procedures for a repository or data management center that prohibit the release of the key to the researchers under any circumstances, until the individuals are deceased; **or**

(c) ☐ There are other legal requirements prohibiting the release of the key to the researchers, until the individuals are deceased; **and**

1. ☐ Biospecimens are not being used to test the effectiveness of a medical device or as a control in an investigation of an investigational device and the results of the activity are to be submitted to the FDA or held for inspection by the FDA; **and**
2. ☐ The records/images/charts that are being collected for this project are NOT from individuals who are or will become recipients of an FDA regulated product (approved or experimental) or act as a control as directed by a research protocol (and not by medical treatment), and the results are to be submitted to FDA or held for inspection by the FDA.

***Coded:** Per the Department of Health and Human Services (HHS) guidance on Engagement of Institutions in Human Subjects Research (2008) **coded** means that: (a) identifying information (such as name or social security number) that would enable the investigator to readily ascertain the identity of the individual to whom the private information or specimens pertain has been replaced with a number, letter, symbol, and/or combination thereof (i.e., the code); and (b) a key to decipher the code exists, enabling linkage of the identifying information to the private information or specimens.

**IF YOUR PROPOSED ACTIVITY MATCHES ONE OR MORE OF THE AFOREMENTIONED ACTIVITIES, IT IS NOT UNDER THE PURVIEW OF THE IRB.**

**IF NONE OF THE ABOVE APPLY TO YOUR ACTIVITY, CONTINUE TO SECTION 2 BELOW.**

**SECTION 2. DETERMINATION OF “RESEARCH”**

**Research**: The HHS definition of research is “a systematic investigation, including research development, testing and evaluation, designed to develop or contribute to generalizable knowledge.”

**2.1** **Is the activity a systematic investigation?**

- **Systematic** means having or involving a system, method, or plan for analyses (e.g., a protocol grant proposal, work statement). A systematic approach is one that involves a pre-determined system, method, plan, and/or procedure(s) to study a specific topic, answer a question, test a hypothesis or develop a theory.
- **Investigation** means a searching inquiry for facts; detailed or careful examination (e.g., hypothesis).

☐ YES

☐ NO

**2.2.** **Is the activity designed to develop or contribute to generalizable knowledge?**

- **Designed** means the study methods are adequate to answer the research question.
- **Generalizable** means universally or widely accepted to apply to a population **beyond** the site or population studied. Research results are expected to contribute to generalizable knowledge by filling a gap in scientific knowledge, or supporting, refining, or refuting results from other research studies.
- **Knowledge** means conclusions expressed for example in theories, principles, and statements or relationships in a particular discipline or body of knowledge.
- ***Note****: Masters’ theses and PhD dissertations are considered to represent generalizable knowledge.*

☐ YES

☐ NO

**IF THE RESPONSE TO QUESTION 2.2. IS “NO”, THE PROPOSED ACTIVITY DOES NOT MEET THE HHS DEFINITION OF RESEARCH; THUS, IT IS NOT UNDER THE PURVIEW OF THE IRB.**

**IF THE RESPONSE TO 2.2 IS “YES”, CONTINUE TO SECTION 3 BELOW.**

**SECTION 3. DETERMINATION OF “HUMAN SUBJECT”**

**Human Subject**: The HHS definition of human subject is “a living individual about whom an investigator (whether professional or student) conducting research: (i) Obtains information or biospecimens through intervention or interaction with the individual, and uses, studies, or analyzes the information or biospecimens; or (ii) Obtains, uses, studies, analyzes, or generates identifiable private information or identifiable biospecimens”

**3.1. Does the project involve LIVING individuals or their biospecimens?**

☐ YES: **CONTINUE TO QUESTION 3.2.**

☐ NO: Is this a genetic study and the data obtained may affect “secondary” participants e.g., family members?

1. ☐ YES: **CONTINUE TO QUESTION 3.2.**
2. ☐ NO: Explain

**IF THE RESPONSE IS “NO” TO BOTH OF THE ABOVE QUESTIONS, THE PROPOSED ACTIVITY DOES NOT MEET THE HHS DEFINITION OF HUMAN SUBJECTS; THUS, IT IS NOT UNDER THE PURVIEW OF THE IRB.**

**3.2. Does the project involve obtaining data ABOUT the living individuals?**

- Projects that collect ONLY information about things e.g., institutions, programs, organizations, etc. and do not collect information about a living individual are **not** considered human subjects research. However, if any information about the individual (his/her title, age, opinion, etc.) is also collected, the project **is** considered human subjects research.
- ☐ YES: **CONTINUE TO QUESTION 3.3.**
- ☐ NO: Explain

**IF THE RESPONSE IS “NO” TO QUESTION 3.2, THE PROPOSED PROJECT DOES NOT MEET THE HHS DEFINITION OF HUMAN SUBJECTS; THUS, IT IS NOT UNDER THE PURVIEW OF THE IRB.**

**3.3. Does the project involve collecting data or biospecimens through intervention or interaction with individuals?**

- **Intervention** means both physical procedures by which data are gathered (e.g., venipuncture, blood pressure, heart monitor, etc.) and manipulations of the subject or the subject's environment (watching a movie, playing a video game, listening to music, etc.) that are performed for research purposes.
- **Interaction**: includes communication or interpersonal contact between investigator and subject (e.g., online surveys, phone or in-person interviews, focus group, etc.).

☐ YES

☐ NO: Explain

**3.4. Does the research involve collecting identifiable private information or biospecimens?**

- **Private information** includes information about behavior that occurs in a context in which an individual can reasonably expect that no observation or recording is taking place, and information which has been provided for specific purposes by an individual and which the individual can reasonably expect will not be made public (e.g., medical record, student records, etc.).
- **Individually Identifiable:** Private information must be individually identifiable (i.e**., the identity of the subject is readily ascertained by the investigator or associated with the private information or biospecimens**) in order for obtaining the information to constitute research involving human subjects.

☐ YES

☐ NO: Explain

**IF THE RESPONSE IS “NO” TO BOTH QUESTIONS 3.3. AND 3.4. , THE PROPOSED ACTIVITY DOES NOT MEET THE HHS DEFINITION OF HUMAN SUBJECTS; THUS, IT IS NOT UNDER THE PURVIEW OF THE IRB.**

**IF THE RESPONSE IS “YES” TO EITHER QUESTIONS 3.3. OR 3.4. , THE PROPOSED ACTIVITY IS HUMAN SUBJECT RESEARCH THAT NEEDS TO BE REVIEWED AND APPROVED BY THE IRB BEFORE IT IS INITIATED.**
