# Supplementary material for: Effects of Patient Portal Message Framing on Treatment Preferences and Expectations for Degenerative Meniscus Tears: Randomized Exploratory Cross-Sectional Survey Study
Source: JMIR Form Res. 2026 May 12;10:e92583. doi: 10.2196/92583 (PMC13213325; doi:10.2196/92583)
Supplement: Supplementary file 2 [file formative_v10i1e92583_app1.docx]

Survey on Physician Language and Preferred Treatment

Start of Block: Default Question Block

Q1 This is a research survey about health-related decision making. Your participation is voluntary. You may stop at any time. Risks are minimal (i.e. you may experience mild discomfort answering health-related scenarios). No personal medical advice is provided in this survey. No personal or health information will be collected. Your responses are anonymous.

- I agree to participate  (1) 

- I do not agree  (2) 

Skip To: End of Survey If This is a research survey about health-related decision making. Your participation is voluntary.... = I do not agree

End of Block: Default Question Block

Start of Block: Block 1

Q6 Are you 18 years of age or older?

- Yes  (1) 

- No  (2) 

Skip To: End of Survey If Are you 18 years of age or older? = No

Q7 Do you currently live in the United States

- Yes  (1) 

- No  (2) 

Skip To: End of Survey If Do you currently live in the United States = No

Q8 Are you confident in your ability to read and answer questions in English?

- Yes  (1) 

- No  (2) 

Skip To: End of Survey If Are you confident in your ability to read and answer questions in English? = No

Q9 Do you currently work in healthcare (i.e. physician, physician assistant, nurse practitioner, nurse, radiology technician, physical therapist, medical student, etc.)?

- Yes  (1) 

- No  (2) 

Skip To: End of Survey If Do you currently work in healthcare (i.e. physician, physician assistant, nurse practitioner, nur... = Yes

End of Block: Block 1

Start of Block: Block 2

Q11 Which of the following apply to you? (Please select all that apply)

- I have had knee pain lasting more than 4 weeks  (1) 

- I have had a knee MRI  (2) 

- I have done physical therapy for knee pain  (3) 

- I have had knee surgery  (4) 

- None of the above  (5) 

Q13 Please indicate how much you agree with each statement.

|  | Strongly disagree (1) | Disagree (2) | Neutral (3) | Agree (4) | Strongly agree (5) |
| --- | --- | --- | --- | --- | --- |
| If a problem could be treated with or without surgery, I prefer to avoid surgery if possible. (1) |  |  |  |  |  |
| If a test shows something is wrong, I would prefer to fix it quickly with surgery rather than wait (2) |  |  |  |  |  |
| I worry about surgery complications (3) |  |  |  |  |  |
| I trust physical therapy as a way to fix a muscle or bone problem (4) |  |  |  |  |  |
| I worry that if surgery is delayed for some kind of issue in favor of physical therapy, that the issue might get worse (5) |  |  |  |  |  |

Q15 How often do you need assistance in reading or understanding materials from a doctor's office or pharmacy?

- Never  (1) 

- Rarely  (2) 

- Sometimes  (3) 

- Often  (4) 

- Always  (5) 

Q16 On a scale from 0-10, how willing are you to take risks? (0 = Not willing at all; 10 = Very willing)

|  | Not willing | Willing | Very willing |
| --- | --- | --- | --- |

|  | 0 | 1 | 2 | 3 | 4 | 5 | 6 | 7 | 8 | 9 | 10 |
| --- | --- | --- | --- | --- | --- | --- | --- | --- | --- | --- | --- |

| Click to write Choice 1 () |  |
| --- | --- |

Q33 When choosing a doctor (or specialist), how important are online reviews/ratings (e.g. Google, Healthgrades, Zocdoc, etc) to you?

- Not important at all  (1) 

- Somewhat important  (2) 

- Moderately important  (4) 

- Very important  (5) 

End of Block: Block 2

Start of Block: ARM_A

Q18 In this scenario, imagine you are a 60-year-old adult who has had knee pain and swelling for nearly 6 weeks. The symptoms started after a twisting movement on that leg and are felt most on the inner side of the knee. The pain is worse when bending the knee, going up or down stairs, and when squatting. You can still walk, but you have had to limit the amount of exercise you do because of the pain and swelling. The knee has not locked in place (never gets stuck when trying to fully straighten the leg), but it sometimes clicks. You were referred to a joint specialist doctor who ordered an MRI of your knee. The MRI report comes back and reads:

MRI Impression: "Horizontal tear of the posterior horn of the medial meniscus."

Your joint specialist later sends you a message that reads: “Hi. Your knee MRI results are back. It shows you have a meniscus tear. Come to the office next week so we can discuss next steps.”

**KEY Context:
- Magnetic resonance imaging (MRI) is a type of medical imaging that is used to take pictures/scans of your body to assess for any injuries or issues.
- The meniscus is a piece of cartilage in the knee that helps with cushioning and stability.

End of Block: ARM_A

Start of Block: ARM_B

Q15 In this scenario, imagine you are a 60-year-old adult who has had knee pain and swelling for nearly 6 weeks. The symptoms started after a twisting movement on that leg and are felt most on the inner side of the knee. The pain is worse when bending the knee, going up or down stairs, and when squatting. You can still walk, but you have had to limit the amount of exercise you do because of the pain and swelling. The knee has not locked in place (never gets stuck when trying to fully straighten the leg), but it sometimes clicks. You were referred to a joint specialist doctor who ordered an MRI of your knee. The MRI report comes back and reads:

MRI Impression: "Horizontal tear of the posterior horn of the medial meniscus."

Your joint specialist later sends you a message that reads: “Hi. Your knee MRI results are back. It shows a meniscus tear that looks more consistent with wear-and-tear changes, common with aging. Come to the office next week so we can discuss next steps.”

**KEY Context:
- Magnetic resonance imaging (MRI) is a type of medical imaging that is used to take pictures/scans of your body to assess for any injuries or issues.
- The meniscus is a piece of cartilage in the knee that helps with cushioning and stability.

End of Block: ARM_B

Start of Block: ARM_C

Q16 In this scenario, imagine you are a 60-year-old adult who has had knee pain and swelling for nearly 6 weeks. The symptoms started after a twisting movement on that leg and are felt most on the inner side of the knee. The pain is worse when bending the knee, going up or down stairs, and when squatting. You can still walk, but you have had to limit the amount of exercise you do because of the pain and swelling. The knee has not locked in place (never gets stuck when trying to fully straighten the leg), but it sometimes clicks. You were referred to a joint specialist doctor who ordered an MRI of your knee. The MRI report comes back and reads:

MRI Impression: "Horizontal tear of the posterior horn of the medial meniscus."

Your joint specialist later sends you a message that reads: “Hi. Your knee MRI results are back. It shows you have a meniscus tear, which means there is damage to the knee's cartilage cushion. Come to the office next week so we can discuss next steps.”

**KEY Context:
- Magnetic resonance imaging (MRI) is a type of medical imaging that is used to take pictures/scans of your body to assess for any injuries or issues.
- The meniscus is a piece of cartilage in the knee that helps with cushioning and stability.

End of Block: ARM_C

Start of Block: Block 7

Q15 To ensure you are paying attention, please select "Somewhat agree" below.

- Strongly disagree  (1) 

- Somewhat disagree  (2) 

- Neither agree nor disagree  (3) 

- Somewhat agree  (4) 

- Strongly agree  (5) 

Q16 When you see the word "degenerative" in a medical report, what does it most likely mean to you?

- Caused by aging / wear over time  (1) 

- Caused by serious infection  (2) 

- Caused by cancer  (3) 

- Not sure  (4) 

Q17 Based on the scenario and MRI results, which treatment option would you prefer and choose as the next step?

- Start physical therapy first  (1) 

- Rest and reassess at a later time  (2) 

- Ask about steroid injection in knee to the reduce pain  (3) 

- Meet with the joint specialist to ask about surgery  (4) 

- Seek a second opinion immediately  (5) 

Q19 Based on the scenario and MRI results, how likely would you be to choose surgery within the next 3 months?

- Extremely unlikely  (1) 

- Somewhat unlikely  (2) 

- Neither likely nor unlikely  (3) 

- Somewhat likely  (4) 

- Extremely likely  (5) 

Q20 Based on the scenario and MRI results, how likely would you be to choose physical therapy before considering surgery?

- Extremely unlikely  (1) 

- Somewhat unlikely  (2) 

- Neither likely nor unlikely  (3) 

- Somewhat likely  (4) 

- Extremely likely  (5) 

Q21 How severe does this knee problem seem?

- Not severe at all  (1) 

- Somewhat severe  (2) 

- Severe  (3) 

- Very severe  (4) 

Q22 How urgently does this knee problem need to be addressed or treated?

- Not urgent at all  (1) 

- Somewhat urgent  (2) 

- Urgent  (3) 

- Very urgent  (4) 

Q23 How confident do you feel in the treatment choice you selected?

- Not confident  (1) 

- Somewhat confident  (2) 

- Confident  (3) 

- Very confident  (4) 

Q24 How much improvement would you expect from each option?

|  | No improvement (1) | Little improvement (2) | Moderate improvement (3) | Great improvement (4) |
| --- | --- | --- | --- | --- |
| Physical therapy (1) |  |  |  |  |
| Surgery (2) |  |  |  |  |
| Rest and reassess later (3) |  |  |  |  |
| Steroid injection in knee (4) |  |  |  |  |

Q25 How risky do you think each treatment option is?

|  | Not risky (1) | Somewhat risky (2) | Risky (3) | Very risky (4) |
| --- | --- | --- | --- | --- |
| Physical therapy (1) |  |  |  |  |
| Surgery (2) |  |  |  |  |
| Rest and reassess later (3) |  |  |  |  |
| Steroid injection in knee (4) |  |  |  |  |

End of Block: Block 7

Start of Block: Block 8

|  |
| --- |

Q26 What drove you to choose your preferred next treatment step? (Select up to 3)

- The word "tear" sounded serious  (1) 

- The joint specialist made it seem urgent  (2) 

- The joint specialist made it seem less serious, common, or age-related  (3) 

- I felt the joint specialist implied surgery as the recommended next step  (4) 

- I want the fastest pain relief possible  (5) 

- I want the fastest return to normal activity and exercise  (6) 

- I worry the knee could get worse if I delay definitive surgical treatment  (7) 

- I prefer to try the least invasive option first  (8) 

- I prefer to quickly fix the issue  (9) 

- I worry about surgical complications  (10) 

- I worry physical therapy won't work or will take too long  (11) 

- Cost or insurance concerns influenced my choice  (12) 

- I don't have the time or ability to attend physical therapy (schedule/transportation)  (13) 

Q27 When you see "meniscal tear," how strongly do you associate it with something that needs surgery to fix?

- Not at all  (1) 

- Somewhat strongly  (2) 

- Strongly  (3) 

- Very strongly  (4) 

Q37 In your follow-up visit with the joint specialist doctor, how would you feel if the doctor disagreed with your preferred next treatment steps?

- Dissatisfied  (1) 

- Somewhat dissatisfied  (2) 

- Neutral  (3) 

- Satisfied  (4) 

- Very satisfied  (5) 

End of Block: Block 8

Start of Block: Block 9

|  |
| --- |

Q28 Please enter your age in years

________________________________________________________________

Q29 What is your gender?

- Male  (1) 

- Female  (2) 

- Non-binary / third gender  (3) 

- Prefer not to say  (4) 

Q38 What is your race?

- White  (1) 

- Black or African American  (2) 

- American Indian or Alaska Native  (3) 

- Asian  (4) 

- Native Hawaiian or Pacific Islander  (5) 

- Other  (6) 

Q31 What is your education level?

- Less than high school  (1) 

- High school graduate  (2) 

- Some college  (3) 

- Associate degree  (4) 

- Bachelor's degree  (5) 

- Graduate/professional degree  (6) 

- Prefer not to say  (7) 

Q32 What is your insurance status?

- Insured  (1) 

- Not insured  (2) 

- Prefer not to say  (3) 

End of Block: Block 9

Start of Block: Block 10

Q33 This survey examined whether wording used in MRI results influences how people may interpret their findings and what treatments they prefer. The scenario provided was hypothetical and is not medical advice. If you have any medical concerns, please consult a licensed physician.

Q34 **Your completion code is: ORTHO-MRI-2025**

End of Block: Block 10
